# Shared Protein Complex Subunits Contribute to Explaining Disrupted Co-occurrence

**DOI:** 10.1371/journal.pcbi.1003124

**Published:** 2013-07-18

**Authors:** Adrian Schneider, Michael F. Seidl, Berend Snel

**Affiliations:** 1 Theoretical Biology and Bioinformatics, Utrecht University, Utrecht, The Netherlands; 2 Centre for BioSystems Genomics, Wageningen, The Netherlands; University of Zurich and Swiss Institute of Bioinformatics, Switzerland

## Abstract

The gene composition of present-day genomes has been shaped by a complicated evolutionary history, resulting in diverse distributions of genes across genomes. The pattern of presence and absence of a gene in different genomes is called its phylogenetic profile. It has been shown that proteins whose encoding genes have highly similar profiles tend to be functionally related: As these genes were gained and lost together, their encoded proteins can probably only perform their full function if both are present. However, a large proportion of genes encoding interacting proteins do not have matching profiles. In this study, we analysed one possible reason for this, namely that phylogenetic profiles can be affected by multi-functional proteins such as shared subunits of two or more protein complexes. We found that by considering triplets of proteins, of which one protein is multi-functional, a large fraction of disturbed co-occurrence patterns can be explained.

## Author Summary

Every genome of current day species contains a very unique selection of genes. Why a specific genome is composed of exactly those genes is determined by many factors, but often not resolvable. It seems plausible that interacting genes would either occur together or be absent together, because if one of them is alone, it might not be able to perform its function properly, just as a bolt can only perform its function together with a nut and vice versa. However, it turns out that interacting genes very often do not nicely co-occur across a wide range of species, and frequently one gene can be found but the other not. In this study, we investigated the co-occurrences of multi-functional proteins and found that they are often maintained in a genome, even if one of their interaction partners is lost. This is because they can still perform some functions with other interaction partners that are still present. We can show that this has a noticeable effect on genome compositions and can explain otherwise surprisingly mismatching co-occurrence patterns of interacting genes.

## Introduction

The gene content of present-day genomes reflects their evolutionary history during millions of years. It is mainly the result of gene gains, duplications, losses and (horizontal) transfer leading to a diverse distribution of genes observed in diverse extant taxa. Common ancestry is a major determinant of the gene content of related species: closely related species generally share more genes than distant ones [1], [2].

However, the correspondence between gene content and phylogeny is not perfect: distantly related genomes share genes if their products are necessary to mediate a defined function or a common lifestyle. This “functional signature of gene content” is exemplified by genes encoding proteins needed for flagellum mediated locomotion: some distantly related eukaryotes share these genes because they have a motile life style or stage, while amongst much closer related organism, some are flagellated and contain flagellar related genes, while others are not flagellated and thus do not have these genes [3]. In general it has been shown that genes with similar, but not merely phylogenetic driven, presence and absence (co-occurrence) patterns form pathways or complexes [4]. This second signal in gene content is strong enough that the similarity of occurrence across genomes can be used to predict interactions; i.e. genes whose phylogenetic distributions are significantly similar have a high probability to encode interacting proteins (e.g. [4]).

However, the reverse does not hold: only 46% of groups of interacting proteins (complexes or modules) were found to have co-occurrence that is better than expected by chance [5]. This trend is even clearer when interactions of protein pairs instead of groups are analysed: In prokaryotes only 24% of interacting protein pairs were found to significantly co-occur [6]. This observation is not caused by technical errors as filtering on such errors has little impact on the percentages [5]. This disrupted co-occurrence of interacting pairs has been frequently reported in small-scale studies where gene presence/absence is the result of manual analysis thereby minimalizing technical errors [7], [8]. For example, a recent analysis of the presence of orthologs of the anaphase-promoting complex (APC) subunits (a crucial protein complex for the progression of the eukaryotic cell cycle) across a diverse array of eukaryotic genomes revealed many genomes with partial (and hence possibly non-functional) protein complexes [7]. A generalized functional or evolutionary explanation for this common pattern has not been tested. Besides, a single unifying explanation is not expected and likely a multitude of functional and evolutionary mechanisms play a role. Importantly, *a priori* perfect co-occurrence is only expected if the the function of a protein is completely functionally dependent on a single other protein and vice versa. Given the complexity of a cell this is likely most often not true. Therefore, we want to investigate the effect of multiple dependencies, i.e. multiple independent interactors, on the absence of co-occurrence.

The importance of proteins with multiple functions (called “moonlighting”) which fulfil different tasks in distinct pathways or complexes is increasingly recognised [9]. This is most clearly defined when focussing on protein complexes where a dynamic view of their composition in different cellular context mediated by shared complex subunits has been established [10]–[14]. In addition moonlighting proteins are continuously being discovered in small-scale molecular biological research such as the succinate dehydrogenase subunit SDH3, which was recently shown to be a component of the TIM22 mitochondrial inner membrane protein insertion complex [15].

**Figure 1:**
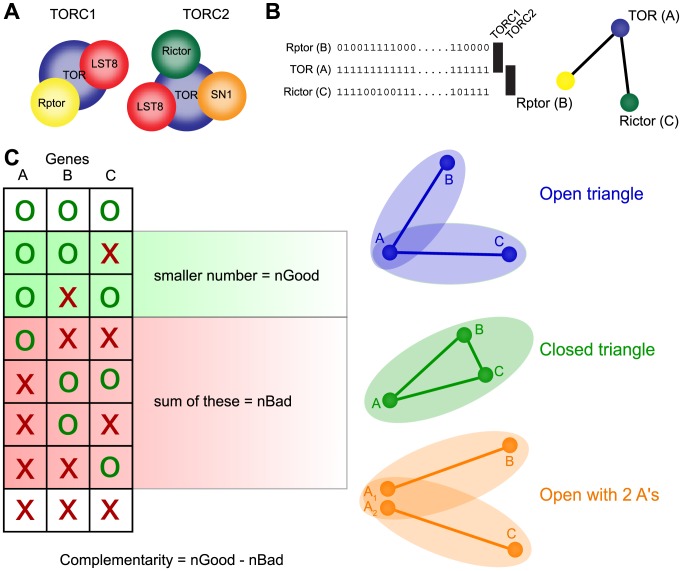
Visualization of complementarity score and illustration of triangle types. (A) Constitution of the two TOR sub-complexes TORC1 and TORC2 complex. (B) Phylogenetic profiles (as an example) of TOR and Rptor and Rictor and the formed triangle. The nodes represent proteins and the lines indicate a pairwise interaction. (C) Visualization of the complementary score and the different triangle types used throughout the study. We consider “open” and “closed” triangles and closed triangles with 2 inparalogs. The ellipses symbolize the different complexes.

Despite awareness of the functional importance of shared interaction partners between complexes or pathways, its impact on genome evolution (presence or absence of genes) has not been widely recognised or researched. However, this functional organisation seemingly provides an intuitive reason for lack of co-occurrence: the absence of an interaction partner makes sense if another interaction partner from another function or context is still present. The target of rapamycin (TOR) complex, a major regulator of growth in eukaryotes, provides an illustrative example: the TOR complex consists of two sub-complexes (TORC1 and TORC2), which are both absent in a distinct but overlapping small set of eukaryotes (Figure 1A) [16]. This is reflected in the complementary co-occurrence patterns of the genes encoding the associated proteins. More formally, this situation can be described by a triplet of proteins (open triangle) where a central protein interacts with two proteins, which do not interact with each other, and where the occurrence patterns of the two interaction partners complement each other.

We want to test to what extent multifunctional proteins such as shared subunits explain disrupted co-occurrence. Therefore, this study considers triplets of interacting proteins in order to comprehensively investigate the influence of additional interactions on understanding the disrupted co-occurrence between a pair of proteins. Triplets of co-occurrence patterns have been previously used to predict functional relations [17]–[19]. Despite the similarity in framework, our approach has a different goal, namely quantifying the effect of proteins with multiple distinct interactions on disrupted co-occurrence in eukaryotes using experimentally determined interaction networks. Although we focus on protein complexes because these are slightly more clearly defined and better conserved in evolution, we also analyse pairwise protein interactions where multiple functionally independent interactions for a single protein could be arguably more expected.

## Methods

### Interaction data

To detect multi-functional proteins in protein interaction networks, we use physical interactions based on protein complex data sets from human and yeast complemented by generic protein-protein interactions from yeast.

In general co-complex membership is more precisely defined than generic protein-protein interactions, which are often an inclusive category of various types of functional relatedness (and thus increases the number of possible reasons for disrupted co-occurrence). For this study, we also require complex definitions where multi-functional proteins are allowed to be in more than one complex; “islands” of non-overlapping complexes are often over-simplified representations of reality and would not allow for capturing the features we are looking for. These criteria lead to two different data sets of complex definitions: From the Corum database of manually curated protein complexes from mammals [20] we extracted the human data, and from the IntAct database [21], we extracted the yeast complexes as defined by [10]. We used the Gavin definitions of complexes which includes core complexes with their associated modules and attachments. For simplicity, we will refer to the two data sets as “human” and “yeast” complexes, respectively. In order to reduce the data-complexity associated with complexes of different sizes and “shapes” (in terms of internal structures, such as modules and attachments), we consider all pairwise relationships within the complexes. Complexes that have no unique members (i.e. they are completely covered by other complexes) have been removed. This concerned 86 complexes in the human data set, but none in the yeast data set.

The 491 yeast and 1,826 human complexes resulted in 45,412 (yeast) and 31,525 (human) pairs of co-complex proteins. The yeast data set is made up of fewer connected components which are on average almost 5 times larger than in the human data set (see Table 1). Consequently, the yeast proteins are also much more connected than the human data, with 50% more interaction pairs, despite 40% less proteins involved.

**Table 1:** Some statistics of the three interaction data sets.

	yeast-compl.	human-compl.	yeast-inter.
number of complexes	491	1,826	–
number of proteins	1,474	2,446	5,521
number of pairs	45,412	31,525	56,078
average degree	61.6	25.8	20.3
connected components	15	125	2
average component size	98.3	19.6	2760.5

We used BioGRID to contrast our results on complexes to more general proteins interactions. This complementary data set allows us to determine the generality or specificity of the outcomes of our analysis with respect to varying interaction definitions. Since we were focusing on physical interactions, we excluded associations based on genetic studies from our analyses. Out BioGRID data set comprises 5,521 yeast proteins forming 56,078 protein pairs residing in two connected components (see Table 1). Consequently, the average degree is lower compared to the complex data.

### Orthologs and phylogenetic profiles

Phylogenetic profiles represent the presence or absence of a gene and its orthologs across genomes. For our large-scale analysis to explore the shape of present-day genomes we compared pairs and triplets of phylogenetic profiles. For this we selected a set of 51 eukaryotic genomics with respect to phylogenetic diversity, in order to cover all major groups of eukaryotes. This selection reflects a trade-off between genome quality and phylogenetic diversity, which both have been shown to be major determinants in how effective phylogenetic patterns can be used for function prediction [22]. For example the *Naegleria gruberi* genome, although not of perfect annotation and sequence quality, is invaluable as it is a genome from a free-living excavate without reduced proteomic diversity.

The coding sequences were downloaded from various sources (see Supplementary Table S1 for a detailed list), and from each gene only the longest transcript was kept. From these genomes, we computed orthologous clusters using an OMA-like algorithm [23], [24], which we adjusted to the specific requirements for this analysis. If not mentioned otherwise, we follow the OMA algorithm, for which we refer to the respective articles, instead of reformulating everything again. Here we will only list the changes to the original algorithm: the minimal alignment score for potential orthologs was reduced to 130, in order to identify also weaker homology, and the minimal alignment coverage was reduced to 40% in a first clustering step (assembling doubly-connected components, as opposed to cliques in the original OMA algorithm) and then alignments with only 25% sequence coverage were added to the best matching cluster. These values were empirically adjusted in order to maximize inclusion of distant homologs while avoiding excessive clustering of paralogs. This lead to 58,533 orthologous clusters for a total of 644,999 proteins. The intended definition of such clusters is to represent all extant descendants from a single gene in the last common ancestor of eukaryotes; or, for a gene invented later, all descendants of that gene. The orthologous cluster data set is provided as Supplementary Dataset S1.

We also used OrthoMCL to form orthologous clusters [25]. OrthoMCL uses different homology search and a completely different clustering strategy than OMA and should therefore give an indication of the robustness of our results towards technical biases related to the orthology detection (Figures S1, S2, S3, S4). Unless explicitly discussed in the Results sections both independent algorithms show similar results which support our conclusions.

The distribution across species of a particular gene can be represented by its phylogenetic profile, a list of presence and absence of the gene and its orthologs in all species in the data set. Unless mentioned otherwise, we are only considering if some orthologs of the gene are present in a certain species or if no ortholog is present. The number of copies (in-paralogs) is not relevant here. For simplicity, when we refer to the phylogenetic distribution of “a gene”, we mean “a gene and its orthologs”.

### Pairwise profile analysis

In the first part of our analysis, we wanted to establish for our data sets, to what degree interacting proteins co-occur across eukaryotes. The co-occurrence of two proteins (i.e. the correlation of presence and absence across species) can be inferred from their phylogenetic profiles. A simple approach to measure the similarity of profiles (and thus co-occurrence) is counting the number of species where one gene is present and the other not; this is called the “Hamming distance” between two profiles. One of the major problem with this approach, next to others, is the correlation among closely related species: e.g. a single gene loss in an ancestor of animals would lead to the absence of that gene in all animal genomes and would thus increase the Hamming distance by several units, even though it is the result of only one evolutionary event. An even larger drawback with this approach is the treatment of losses in the phylogenetic profile: shared losses count as much towards similarity as shared presences and, given the sparse nature of the data and the large-scale approach, will lead to a certain level of uncertainty in calculating phylogenetic profiles (see e.g. [26]). Thus, better measures have been developed, such as the “partial correlation” used by [27]. It is based on the correlation of reconstructed gene gains and losses on the branches of the species tree, corrected by the correlation with the global trend (e.g. whole-genome duplications or genome streamlining events). This is also our choice for the analyses presented here.

For pairwise profile analysis, we considered only pairs in which at least one of the two genes is present in at least half of all species, in order to ensure that the analysis captures the global trends and only uses gene pairs that actually co-occurred at least at one point during evolution. We defined the conditions under which we can consider two phylogenetic profiles to be very similar, which will be called “matching”: exact equality of the profiles is clearly too stringent, because of the many possible sources of error (e.g. missing gene predictions), but also since a loss in a single species is often not very consequential for the overall picture.

Thus, we used the partial correlations among all protein pairs from the species under study with at least one interaction as the background distribution (of which the vast majority are non-interacting pairs). For our main results, we consider two interacting proteins as having “matching” profiles, if their partial correlation exceeds that of 90% of these background pairs. In addition we report the results for 85% and 95% in Figures S8 and S9 (for both OMA and MCL). Our randomization should correct for other signals in the occurrence data, such as the common ancestry signal in gene content, and the fact that proteins with interactions are better studied and/or more essential which could also impact occurrence patterns. This is a similar strategy as previously employed by [5], [28] to determine whether a group of proteins is exhibiting significant co-occurrence in its evolution. This approach also allows to define a threshold independent of a specific similarity measure.

If pairs are not matching, we also test whether they classify as pairs that are in a subset pattern. Subset patterns are thought to occur for two postulated reasons that create this type of non-matching pattern. The first postulated cause for a subset relation is because there is an assymmetric relation between two proteins, and the second is because one protein was invented much later than the other (lineage specific additions as predicted by the irremediable complexity hypothesis [29]).

First we determine whether one OG is a taxonomic subset of another. An OG is considered to be a taxonomic subset of another OG, if they contain proteins from overlapping species sets, but one of these sets spans a wider taxonomic range. Subsequently we score the general subset-nature of the relation of one OG to the other. Being a subset means that if gene A is present in a species, then gene B may or may not be present, but if A is absent, then B should also be absent. This situation can arise if B depends on A, but A not on B. We quantify this property by counting in how many species A is present without B (cases supporting the subset), minus the number of species where B is present without A (cases violating the subset property), with A being the protein that occurs in more species. Again, we cannot expect many perfect subsets, for the same reasons as described above. Thus, we apply again the rule that two profile are considered to be in a “subset” relationship, if they achieve a higher subset score than 90% of the background set of all protein pairs in the data set. A subset-like relationship can also occur, if one of the genes was invented later, in which case there is no asymmetric dependency between the genes as described above. These are independently tested and classified before as a taxonomic subset even if they would have classified as a subset.

### Triplet analysis

The main focus of our analysis is on protein triplets, in order to quantify the influence of multi-functional proteins on co-occurrence disruptions. To this end we analysed triplets of proteins that all have at least one interaction, but that not necessarily interact with each other. All triplets A, B, C were evaluated with a “complementarity score” which is based on the phylogenetic profiles of the 3 genes and expresses to which degree the following properties are fulfilled: If A is present, then at least one of B and C should also be present in a genome, but many genomes should be missing one of B or C, ideally about equally often. In other word, B and C should complement each other with respect to A, hence the name complementarity score. This score is computed by taking the smaller number of the “good” cases, where in a species either A is present but only B or only C, and subtract from this number the “bad” cases, i.e. the number of species in which the above outlined conditions are violated. The scoring scheme is illustrated in Figure 1 B. As for the pairwise analysis, we were only interested in global trends and thus require the protein A to be present in at least half of all species.

## Results

### Profile comparison of interaction pairs

The main goal of this study is to investigate the amount of disrupted co-occurrence that can be explained by a protein being shared between two complexes, or a protein having multiple distinct interactions. First, however, we want to establish the degree of disrupted co-occurrence in our data sets, i.e. how often co-complex or interacting protein pairs have a similar profile and what other patterns can be observed between non-similar profile pairs.

**Figure 2:**
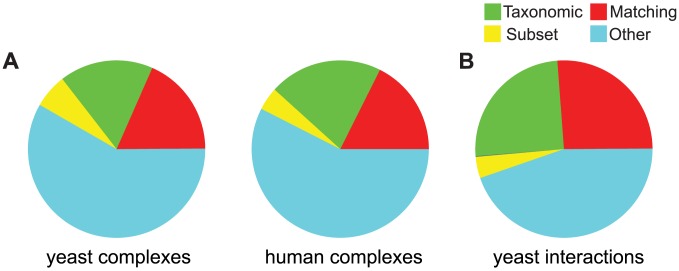
Distribution of the profile relationships of (A) co-complex protein pairs in the two complex data sets and (B) yeast interactions. The 4 differently colored slices correspond to the types of similarities between profiles as described in Methods.

For this we compared the profiles of all pairs of interacting proteins in all three data sets (yeast and human complexes, and yeast interactions) and classified the relationship into four different types as described in Methods. The result, shown in Figure 2, indicates that less than a fifth of interacting proteins also have a matching profile (about 18% in both data sets, red slice). This is in line, albeit slightly lower, than previous findings in prokaryotes [6].

Only a small fraction of protein pairs (6.2% in yeast, 4.2% in human, yellow slice) fulfill the subset relationship, i.e. gene B was lost in some species where A is still present. Using an alternative orthology (OrthoMCL), this number is noticeably higher reflecting the differences in the orthology detection algorithm.

We also found that a relatively large proportion of the pairs (17% in yeast, 21% in human) show a taxonomic pattern, i.e. one of the genes was invented at a later point and is thus not present in the earlier-branching species. This confirms previous observations that phylogeny leaves a strong signal in gene content [1]. Interestingly, both the yeast as well as the human data set lead to similar distributions of the different types of profile relationships as do the interactions and the complex data sets, despite having different network structures (see also Table 1).

These results demonstrate that pairwise analysis is not sufficient to explain the complex relationship between phylogenetic profiles and protein interactions, as there is a large fraction of interacting pairs with no obvious profile pattern (“other”, light blue slice Figure 2). Although a considerable fraction of interacting proteins show a matching profile, mechanisms or scenarios have to be found to explain why interacting proteins often do not display phylogenetic co-occurrence.

### Triplet analysis

We propose that some of the unexplained mismatching profiles could be due to multi-functional proteins. For this we consider proteins that are part of two different complexes, which can lead to scenarios, where if one of the two complexes is (partially) lost, the protein would still be needed to perform its function in the other complex. We also consider proteins that have multiple pairwise interactors which themselves do not interact with each other to contextualize our observations. To investigate this scenario and its effect on phylogenetic profiles, we systematically analyzed the patterns found among profile triplets. Specifically, we investigated the patterns among what we call “open triangles”, where a protein A interacts with both B and C, but B and C are independent of each other (i.e. do not interact, see Figure 1 B for a visualization). In the context of protein complexes, this means that A is a shared subunit of the two complexes containing B and C, respectively.

Since A would still be able to perform part of its function if either B or C were lost, the phylogenetic profiles of open triplets are expected to have the features described by the complementarity score (see above and Figure 1) B, namely that B and C show little co-occurrence, but that both are subsets of A. This means that if we performed only pairwise analysis, the pairs A–B and A–C are, although interacting, expected to have non-matching profiles.

In a first step of the triplet-based analysis, we try to establish if open triangles in the protein complexes and protein interaction data correspond to the expected phylogenetic pattern described above. To this end, we analyzed all triplets of the proteins from the complexes and interaction data sets, and from that collected all open and closed triangles, as well as a random selection of approximately 10 million triplets of proteins that do not interact with each other. All these triplets were scored with the complementarity score as described in the Methods and ranked by that score. Finally, the triplets were divided into equally large bins ranging from the lowest to the highest complementarity score. For each bin, we counted the occurrences of open and closed triangles.

**Figure 3:**
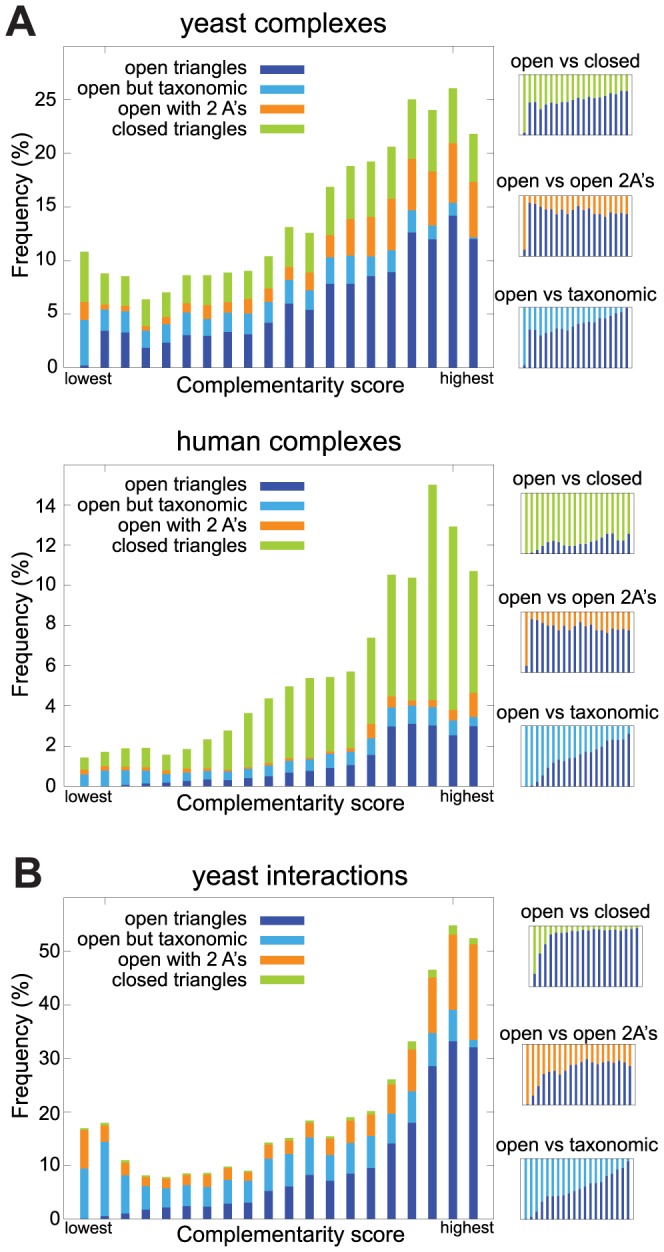
Frequency of open triangles as a function of the triplet score for (A) co-complex protein pairs and (B) yeast interactions. Each bar corresponds to an equally-sized bin, with the right-most bar belonging to the best scoring triplets. The three small plots on the right side of the figures displays ratios between different categories.

The results are summarized as stacked histograms in Figure 3. Each bar represents the distribution of triplet types within a bin, with the left-most bin corresponding to the lowest complementarity score and the right-most bin to the highest score. The bins are of equal size, but the vast majority of triplets are trios of non-interacting proteins (which make up the white space above the colored bars). The graphs show a clear correspondence between complementarity score and the frequency of open triangles (dark blue), indicating that the pattern described by this score often implies, as expected, open triangles. In fact the Spearman *ρ* for the increase in the fraction of open triangles over bins of increasing score is between 0.92 and 0.99 for all three data sets with p<10e-5 (Figures S5, S6, S7). The bars in light blue also correspond to open triangles, but there are cases where B or C are taxonomic subsets of A and thus a different mechanism is probably at work. This is reflected by the fact that especially low-scoring triplets fall into this category. There is also an enrichment among high-scoring triplets of open triangles with two in-paralogs in A (orange bars). This is expected, since this scenario (recent duplication in A and then possibly sub-functionalization of the two in-paralogs) is likely to result in similar phylogenetic patterns to when A did not duplicate. Interestingly, and possibly an indication of the robustness of the results, these features are very similar among the three data sets that we analyzed. The main difference between the data sets is the much larger fraction of closed triangles between the complex data and BioGRID, mainly reflecting the difference in definition of protein pairs. Moreover, the human complex data (green bars) also has a higher fraction of closed triangles compared to the yeast complexes, which is due to the different network structures of the interaction data sets (see Table 1). Closed triangles are also enriched among triplets with a high complementarity score, albeit to a somewhat lesser degree.

It follows from this analysis that open triangles often correspond to the complementary pattern of phylogenetic profiles. Thus, proteins involved in such triangles will be part of interactions where the phylogenetic profiles do not match. In particular the pairs A–B and A–C of open triangles are interacting, but will often have mismatching profiles. Such disrupted co-occurrence would seem unexplainable in pairwise analysis, but when triplets are considered, an explanation can often be found.

### Using triplet information in the pairwise analysis

**Figure 4:**
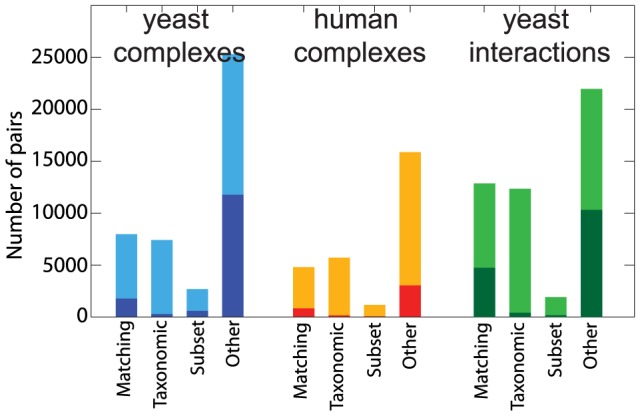
Distribution of profile-pair types among co-complex proteins and yeast interactions. The pairs are divided into the the same categories as in the pie charts of Figure 2. The dark part of each bar corresponds to the number of co-complex protein pairs or yeast interactions that are part of a positive-scoring triplet.

Having established the impact of shared sub-complexes or proteins with multiple distinct interactions on triplets of phylogenetic profiles, we quantified their effect on the disrupted co-occurrence of co-complex pairs reported above. For this we considered all open triangles with a positive complementarity score and extracted all co-complex pairs involved in these triangles. For these pairs we then determined the categories of profile pattern pairs that we also used in Figure 2, i.e. whether they are matching, a subset, taxonomic or undefined (“other”). The result is visualized in Figure 4, where the dark parts of the bars indicate the number of pairs from positive-scoring triangles among all co-complex pairs in the respective category.

In the human data set, 4,123 (15%) of 19,198 co-complex pairs are found in positively scoring triangles, while among the yeast complex data 14,452 (33%) of 43,338 co-complex pairs are part of positively scoring triangles. In correspondence with Figure 3, there are more positive scoring open triangles among the yeast complexes due to the different network structure.

This analysis shows that the majority of pairs from positively scoring triplets fall into the “other” category (3,042 or 74% in human and 11,791 or 82% in yeast). Compared to the expectation under random assignment, this is an over-representation in this category of 40% in yeast and 29% in human, which is modest, but because of the large numbers, highly significant (both p<1e-10). There is also a small fraction of triplet-pairs that are classified as “matching”, as this category also includes profile pairs that are not perfectly equal. In the yeast data set, there are also a few triplet-pairs among the subset and taxonomic categories.

This analysis confirms that for a substantial fraction of co-complex pairs with mismatching profiles, the disrupted co-occurrence can be explained by considering triplet relationships among proteins.

**Figure 5:**
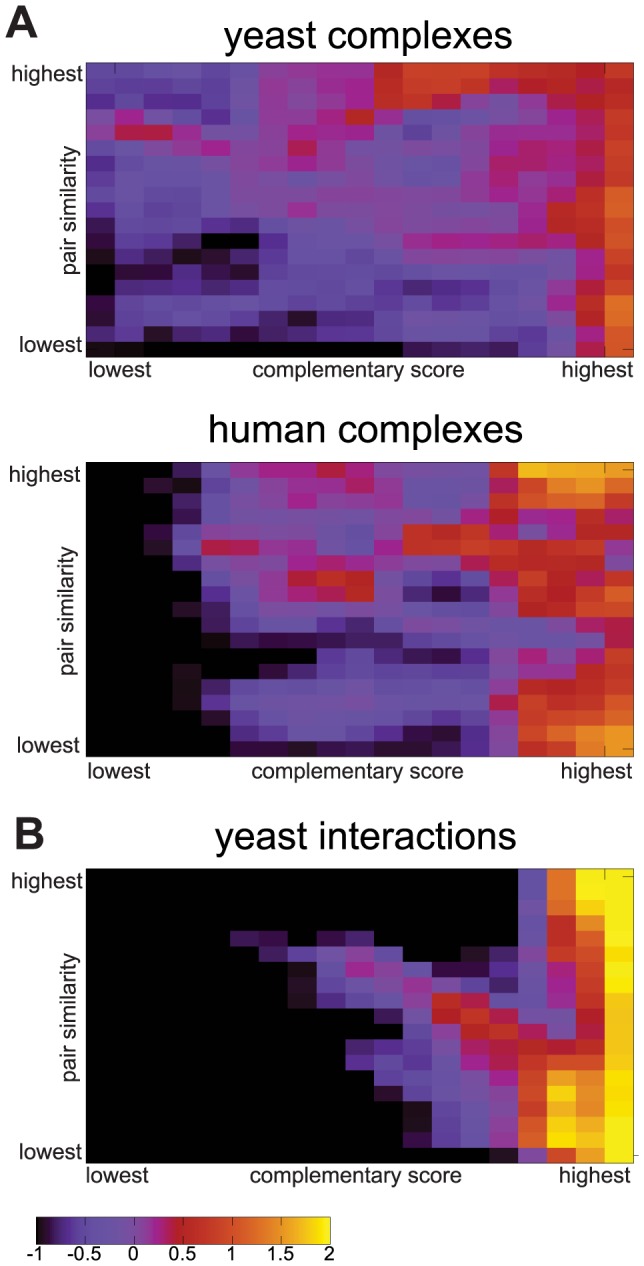
Heat maps showing the enrichment of (A) co-complex pairs or (B) yeast interactions as a function of pairwise profile similarity scores and triplet-based complementarity scores. The color intensity corresponds to the log2-enrichment of protein pairs that are part of the same complex or interact, among all protein pairs inside a pair- and triplet-based bin.

In order to visualize and understand the relation between profile pair similarity (partial correlation), triplet-based complementarity score and interaction probability, we created heat maps that show the enrichment of co-complex pairs or interactions as a function of the two types of scores (Figure 5). Although also a remarkable number of pairs with low pairwise similarity and triplet scores share complex membership or interact, there is a clear trend in all data sets for both measures to correlate with higher enrichments of interactions: The fields with the highest enrichment of co-complex pairs (orange and yellow) tend to be at the top (high pairwise similarity) and right-hand side (high triplet score) of the maps. For the yeast complex data set, the highest complementarity score seems to be a very good indicator for co-complex membership, whereas for the human complexes set, the combination of very high pair similarity and high complementarity score seems to correlate best with the enrichment of co-complex pairs, but also the complementarity score alone is a good predictor of interaction.

This confirms that, as implied by Figure 2, the prediction of interacting pairs based on only pairwise profile similarity will produce many false negatives. Many more co-complex pairs can be predicted by considering also higher-order scenarios, such as the one presented here based on open triangles.

### Examples

A previously studied example of multi-functional protein sub-complexes is the evolution of the target of rapamycin (TOR) complex, a major regulator of growth in eukaryotes. It has been shown that TOR consists of two sub-complexes, but not both of them are present in all eukaryotes. This is reflected in the phylogenetic profiles of the associated proteins, which lead to patterns similar to what we intend to capture with the complementarity score [16]. It is thus of great interest to investigate how TOR behaves in our fully automated large-scale analysis, both in terms of complementarity score as well as accuracy of the phylogenetic profiles.

TOR complex 1 consists of *MTOR*, *MLST8* and *RPTOR*, while TOR complex 2 also contains *MTOR* and *MLST8*, but combined with *RICTOR* and *MAPKAP1*. In Table 2 we show the profiles of the triplet *MTOR*–*RPTOR*–*RICTOR* using our data and orthologs. The phylogenetic profiles indicate that these 3 proteins also show the typical pattern, with *RICTOR* missing in 11 species where *TOR* and *RPTOR* are present. However, *RPTOR* is almost ubiquitous, only missing in 3 species that have both of the other proteins. Together with 4 species violating the complementarity pattern, this results in a slightly negative complementarity score of −1 (which is still among the 10% highest-scoring triplets).

**Table 2:** Examples of triplet profiles.

	TOR complex			Emerin-related		
	*MTOR*	*RPTOR*	*RICTOR*	*HNRNPK*	*PDCD4*	*CDC37*
*Homo sapiens*	▪	▪	▪	▪	▪	▪
*Mus musculus*	▪	▪	▪	▪	▪	▪
*Takifugu rubripes*	▪	□	▪	▪	▪	▪
*Danio rerio*	▪	▪	▪	▪	▪	▪
*Ciona intestinalis*	▪	▪	▪	▪	▪	▪
*Branchiostoma floridae*	▪	▪	▪	▪	▪	▪
*Caenorhabditis elegans*	▪	▪	▪	▪	□	▪
*Drosophila melanogaster*	▪	▪	▪	▪	▪	▪
*Anopheles gambiae*	▪	▪	▪	▪	▪	▪
*Nematostella vectensis*	▪	▪	▪	▪	▪	▪
*Trichoplax adhaerens*	▪	▪	▪	▪	▪	▪
*Monosiga brevicollis*	▪	▪	▪	▪	▪	□
*Batrachochytrium dendrobatidis*	▪	▪	▪	▪	□	▪
*Neurospora crassa*	▪	▪	▪	▪	□	▪
*Yarrowia lipolytica*	▪	▪	▪	▪	□	▪
*Debaryomyces hansenii*	▪	▪	▪	▪	□	▪
*Kluyveromyces lactis*	▪	▪	▪	▪	□	▪
*Candida glabrata*	▪	▪	▪	▪	□	▪
*Saccharomyces cerevisiae*	▪	▪	▪	▪	□	▪
*Schizosaccharomyces pombe*	▪	▪	▪	▪	□	▪
*Cryptococcus neoformans*	▪	▪	▪	▪	□	▪
*Ustilago maydis*	▪	▪	▪	▪	□	▪
*Phycomyces blakesleeanus*	▪	▪	▪	▪	□	▪
*Rhizopus oryzae*	▪	▪	▪	▪	□	▪
*Encephalitozoon cuniculi*	□	□	□	□	□	□
*Entamoeba histolytica*	▪	▪	▪	□	□	▪
*Dictyostelium discoideum*	▪	▪	▪	▪	□	□
*Plasmodium falciparum*	□	□	□	▪	▪	□
*Cryptosporidium parvum*	□	□	□	▪	▪	□
*Toxoplasma gondii*	▪	□	□	▪	▪	□
*Tetrahymena thermophila*	▪	□	▪	▪	□	□
*Paramecium tetraurelia*	▪	□	▪	▪	□	□
*Phytophthora infestans*	▪	▪	▪	▪	▪	□
*Phytophthora sojae*	▪	▪	▪	▪	▪	□
*Phaeodactylum tricornutum*	▪	▪	□	▪	▪	▪
*Thalassiosira pseudonana*	▪	▪	□	▪	▪	▪
*Emiliania huxleyi*	▪	▪	□	▪	▪	▪
*Physcomitrella patens*	▪	▪	□	▪	▪	□
*Arabidopsis thaliana*	▪	▪	□	▪	▪	□
*Oryza sativa*	▪	▪	□	▪	▪	□
*Selaginella moellendorffii*	▪	▪	□	▪	▪	□
*Chlamydomonas reinhardtii*	▪	□	□	▪	▪	□
*Volvox carteri*	▪	□	□	▪	▪	□
*Micromonas pusilla*	▪	▪	□	▪	▪	▪
*Ostreococcus tauri*	▪	▪	□	▪	▪	▪
*Cyanidioschyzon merolae*	▪	▪	□	□	□	□
*Leishmania major*	▪	▪	▪	□	□	□
*Trypanosoma brucei*	▪	▪	▪	□	□	□
*Giardia intestinalis*	▪	□	□	□	□	□
*Naegleria gruberi*	▪	▪	□	□	□	□
*Trichomonas vaginalis*	▪	▪	▪	□	□	□
nGood	min(3,11)			min(13,12)		
nBad	4			4		
**Complementarity score**	**-1**			**8**		

Left: a protein triplet from the TOR complex, right: a triplet from emerin-related complexes. The computation of the complementarity score is explained in Figure 1 B.

Since these profiles stem from large-scale and uncurated analyses, it is possible that the violations of the pattern are mistakes in the data rather than biologically meaningful. Possible error sources include incompletely sequenced or annotated genomes (leading to missing gene predictions), failure to detect weak homology, or problems with the clustering of orthologs. With detailed manual analysis, the fate of some “suspicious” absences might be discovered. But also using just the data from large-scale analysis, the complementarity pattern can be observed in this previously studied example from the TOR complex.

Among the highest scoring open triangles in our data set, the emerin-related complexes 25 and 52 (named on the basis of their S300 elution fraction number, see [30]) were found several times. Emerin-related complexes have been shown to be involved in a variety of different functions. This is achieved via several multi-protein complexes with a small set of proteins that participate in multiple complexes. Since apparently not all functions of these emerin-related complexes are needed in all organisms, the shared subunits are expected to be present in many species, while some of the other proteins are not always needed, and thus can potentially be lost in some species. This is exactly the scenario on which this study focuses and the complementarity score is aimed at revealing cases like those found in emerin-related complexes. The complexes 25 and 52 share a number of proteins, such as *ACTB*, *EMD*, *HNRNPK* or *NAAA38*, while both contain also other proteins. In our example, *HNRNPK* (heterogeneous nuclear ribonucleoprotein K) takes the role of the protein A that is common to both complexes, while *PDCD4* (programmed cell death protein 4) is only part of complex 52 and *CDC37* (cell division cycle 37) is only part of complex 25 [30].

The phylogenetic profiles of these 3 proteins are shown on the right-hand side of Table 2. From only comparing two profiles at a time, interactions among these profile would seem quite unlikely, as they do not seem to be correlated. However, when the whole triplet is considered, we can observe the complementarity pattern as proposed in this study: *PDCD4* and *CDC37* are almost perfect subsets of *HNRNPK*, while normally at least one of the two is present if also *HNRNPK* is found in a genome. According to the genome annotations and the ortholog predictions used here, *PDCD4* is missing in 13 species (among them all fungi) that all have *HNRNPK* and *CDC37*. On the other hand, *CDC37* is missing in 12 species where the other two proteins are present, mostly in plants and chromalveolates. The complementarity criterion is only violated in 4 species, thus this results in a high overall score of 8.

This shows that the proposed scenario of multi-functional protein sub-complexes leaving traces in the phylogenetic profiles is not only a signal found in large-scale comparisons, but that the patterns can be confirmed by well-studied and biologically meaningful examples.

### Duplications of proteins in shared sub-complexes

Not only gene loss and invention shape the content of a genome, but also duplications are important factors. In the context of shared sub-complexes, it has been shown that multi-functional proteins have a larger chance of being retained subsequent to duplications in order to specialize in the different roles they perform in the different complexes [11], . One such example is the above-mentioned TOR complex, where the *MTOR* ortholog was found to be duplicated in several species [16]. In the analysis of the complementary score (Figure 3), the orange parts of the bars show the enrichments of open triangles where gene A is duplicated and the two inparalogs take the different roles in the interaction. Just as the normal open triangles, there is also a strong enrichments of triangles with duplications among the high-scoring triplets.

Our data set allows for a more explicit, large-scale test of the hypothesis that multi-functional proteins tend to be retained after duplication to a higher extent. To this end, we analyzed the rate of which duplications are retained (‘duplication rate’) of the proteins with different roles among the various triplet types of this study. This rates were computed from the number of proteins with duplications (defined as having in-paralogs in at least 5 species) among all proteins of which at least 5 species have only one copy. This filter excludes some proteins from the analysis, but reduces the chance of including clusters with out-paralogs or accidental duplication calls because of misannotations.

The results are shown in Table 3 for various classes of proteins, computed using the human complexes (using the yeast complexes yielded very similar results, data thus not shown). For open triangles, the clear difference between the A protein, which is shared among both complexes, and the B and C proteins, which are only in one complex, can be observed: 88.4% of the A's are duplicated, but only 66.3% of B and C underwent duplication. Because of the very large numbers of proteins involved, this difference is highly significant (p<1e-50, *χ* ^2^-test). Also, the duplication rate of B and C may seem high, but it is in accordance with previous estimates of duplication rate (see e.g [33]), and also very close to the “background” rate of 64.8% found in non-connected triplets. Unsurprisingly, among the open triangles where A is already known to be duplicated and specialized, 100% of all A's underwent duplications according to the above definition, while only 64.2% of the two other proteins have duplicated.

**Table 3:** Percentages of duplicated genes among different categories.

		percent duplicated	
Category	N	A	B or C
Open triangles	172,592	88.4	66.3
Open with 2 A's	13,863	100.0	64.2
Closed triangles	346,401	75.9	
Other triplets	7,937,367	64.8	

Percentages of duplicated genes among different categories. N is the number of triangles or triplets. Column “A” gives the percentage of genes with duplications in gene A (the shared subunit), while column “B or C” gives the percentage of duplications of the other two genes. For the closed triangles and other triplets (with no direct interactions), there is no central gene A and thus all 3 genes were counted as the same category.

These results confirm previous observations that proteins shared among complexes tend to be retained more often after duplication. It also shows that in genome evolution, not only gene loss and gain, but also duplications are significantly influenced by shared complex subunits.

## Discussion

In this study, we only analyzed the case of shared complex subunits. This was sufficient to allow explanations for a considerable fraction of the non-matching profile pairs. However, there are likely other interaction scenarios, possibly even higher-order than just triplets, that could explain even more of the seemingly unexpected profile pair relationships. Unfortunately, in higher-order analyses, such as on quadruples, many of the problems like missing gene predictions, imperfect orthology assignments etc, are magnified, making it even harder to distinguish the true signal from noise or bias.

A complementarity pattern for a triplet of interacting genes could be caused by technical reasons instead of shared subunits or multifunctional proteins. Such technical reasons as failed gene predictions or missed orthologs leading to complementary absences can also cause a lower matching but a higher complementarity score. We think that such technical errors are an important potential reason why the number of closed triangles increases with a high complementarity score. Conversely our open triangle interaction patterns as derived from the protein complex (and interaction) databases, can be interpreted in functional terms in a more complicated manner. Firstly a single shared subunit between two otherwise unrelated complexes forms just one side of a possible continuum. At the other extreme would reside a complex which functions in two so called “complex variants,” where A (together with other core proteins) attach either B or C to a core complex. Our bioinformatic analyses treats both cases the same, and thus the latter scenario is likely also present in our analysis.

Our analysis adds to the growing list of scenarios where disrupted co-evolution can be explained by biological processes. One example outside protein complexes are asymmetric relationships within metabolic pathways. It has been shown that if a protein A depends somehow on another protein B, but not vice versa, then phylogenetic patterns similar to the “subset” category of our analyses can be found [19]. In this study, we focused on protein complexes, where asymmetric relationships are expected to a much lesser extent. However, the “subset” pattern is still widespread among the involved proteins, and in many cases can be explained by the presence of other complexes that share subunits.

We also found that a large fraction of the profiles of co-complex pairs fall into the “taxonomic” category, which means that one of the two proteins was invented later and thus has a taxonomically more limited range. As these situations might lead to patterns similar to those captured by the complementarity score (especially if B and C were invented in different lineages), we had to treat them separately in our analyses. Nonetheless, the high frequency of these taxonomic additions is notable and would deserve some explanation. An interesting recently proposed hypothesis is the “irremediable complexity” theory [29] that proposes a mechanism by which complexes would increase in size during evolution: if a new gene joins an existing complex in a nearly neutral fashion but after accumulating correlated substitutions becomes inseparable from the complex, it will have a high probability of being retained. This is obviously a very different evolutionary process than the scenario with shared subunits which we studied here.

Fundamental biological question such as why the human or any other genome contains the combination of genes that it does, are far from being answered. In this study, we tested a possible explanation for a related problem, namely why some proteins are retained in a genome despite the absence of their interaction partner. Our findings show that proteins that participate in more than one complex are often maintained in the genome even after a co-complex protein has been lost. This effect plays an important role in explaining disrupted co-occurrences or incomplete complexes in sequenced but poorly studies genomes, where analyses based on only a pair of proteins are not sufficient to resolve the evolutionary mechanisms.

## Supporting Information

Dataset S1 **Orthologs.** Definitions of the orthologous clusters used in this analysis. The zip file contains two plain-text tables: the cluster definitions using internal IDs (consisting of 4-letter species code and gene number) and a mapping of the internal IDs to various identifier formats as used in the source genome files.
(ZIP)

Figure S1 Distribution of the profile relationships of co-complex protein pairs in the two complex data sets (A–D) and yeast interactions (E–F) for the OMA and MCL algorithm.
(EPS)

Figure S2 Frequency of open triangles as a function of the triplet score for co-complex protein pairs (A–D) and yeast interactions (E–F) for the OMA and the MCL algorithm.
(EPS)

Figure S3 Heat maps showing the enrichment of co-complex pairs (A–D) or yeast interactions (E–F) as a function of pairwise profile similarity scores and triplet-based complementarity scores for the OMA and the MCL algorithm.
(EPS)

Figure S4 Distribution of profile-pair types among co-complex proteins (A–B) and yeast interactions (C) for the OMA and the MCL algorithm.
(EPS)

Figure S5 Spearman correlation (*ρ* and p-value) for the fraction of different triangle types over bins for the yeast interactions.
(EPS)

Figure S6 Spearman correlation (*ρ* and p-value) for the fraction of different triangle types over bins for the human complexes.
(EPS)

Figure S7 Spearman correlation (*ρ* and p-value) for the fraction of different triangle types over bins for the yeast complexes.
(EPS)

Figure S8 Classification of pairwise profiles for the three interaction data sets under different cutoffs using the OMA algorithm.
(EPS)

Figure S9 Classification of pairwise profiles for the three interaction data sets under different cutoffs using the MCL algorithm.
(EPS)

Table S1 Genomes list. Complete list of all 51 genomes, with species name, 4-letter abbreviation, data source and retrieval date.
(XLS)
